# Bortezomib‐based therapy is effective and well tolerated in frontline and multiply pre‐treated Waldenström macroglobulinaemia including BTKi failures: A real‐world analysis

**DOI:** 10.1002/jha2.597

**Published:** 2022-10-20

**Authors:** Jahanzaib Khwaja, Encarl Uppal, Robert Baker, Kajal Trivedi, Ali Rismani, Rajeev Gupta, Ian Proctor, Charalampia Kyriakou, Shirley D'Sa

**Affiliations:** ^1^ Department of Haematology University College London Hospitals London UK; ^2^ Health Services Laboratories London UK; ^3^ Department of Cellular Pathology University College London Hospitals London UK

**Keywords:** bortezomib, BTKi, Waldenström macroglobulinaemia, lymphoplasmacytic lymphoma

## Abstract

Waldenström macroglobulinemia (WM) is a rare, incurable low grade lymphoma following a relapsing trajectory. Management strategies have evolved with the introduction of targeted therapy including new classes of Bruton tyrosine kinase inhibitor (BTKi). Treatment may however be limited particularly at relapse by a lack of drug availability and tolerability. We assessed the real‐world efficacy and tolerability of bortezomib‐containing regimens in patients with WM at frontline and relapse including those with prior BTKi resistance. Forty‐one patients were identified with 44 bortezomib‐containing regimens administered (*n* = 12 frontline, *n* = 32 relapse). Of patients treated at relapse, the median prior lines of therapy was 3 (range 1–7). 24% (10/41) of the cohort were refractory or intolerant to BTKi prior to bortezomib delivery. The median follow‐up after bortezomib administration was 34 months (range 0‐131). Overall response rate was 88%; 2‐year overall survival and progression‐free survival were 90% (95% confidence interval [CI] 73–96) and 76% (95% CI 55–87), respectively. Median time‐to‐next‐treatment was 66 months. Neuropathy (grade 1–2) occurred in 24% (8/34) and did not result in treatment cessation in any case. Gastrointestinal disturbance occurred in 7% (3/41). Treatment discontinuations were rare (1/44; 2%), suggesting a manageable safety profile. Major response rate was comparable in those with prior BTKi compared with those without (75% [6/8] vs 84% [27/32], *p* = 0.61). Bortezomib should be considered as a treatment modality particularly in those who are refractory to BTKi.

## 1 BACKGROUND

Waldenström macroglobulinemia (WM) is a rare incurable low grade lymphoma, which typically follows a prolonged disease course with a remitting and relapsing trajectory. Treatment may be of fixed‐duration with rituximab‐containing chemoimmunotherapy combinations (such ascyclophosphamide, bendamustine or bortezomib‐containing regimens) or continuous with Bruton tyrosine kinase inhibitors (BTKi). Treatment selection is based on disease characteristics, prior therapy, local availability and patient preference. Treatment options may be limited at relapse by a lack of therapeutic agent available and drug tolerability.

Bortezomib is a proteasome inhibitor that has been used as a standard regimen for multiple myeloma and established in systemic AL amyloidosis. It does not cause significant myelosuppression and does not require dose adjustments for renal impairment. Neuropathy is the primary and limiting toxicity and a particular concern in early trials in WM [1]. In those with multiple myeloma, bortezomib has been shown to have equal efficacy on a weekly rather than twice‐a‐week schedule and subcutaneous rather than intravenous with additional reduced rates of peripheral neuropathy [2, 3]. It has been used in prospective phase II frontline [1, 4, 5] and relapsed [6, 7] trials prior to the widespread use of BTKi.

We assessed the efficacy and tolerability of bortezomib‐containing regimens in patients with WM at frontline and relapse in the real‐world setting including those with prior BTKi exposure.

## 2 METHODS

Adult patients (≥18 years) who received a subcutaneous bortezomib‐containing regimen for WM between 2010 and 2022 across six centres in the United Kingdom were retrospectively reviewed. Data were acquired from the national Rory Morrison Registry. Research ethics was obtained. Adverse events were graded in accordance with National Cancer Institute Common Terminology Criteria for Adverse Events version 5.0 and response assessed according to IWM response criteria. Overall survival (OS) and progression‐free survival (PFS) were calculated as the time from first bortezomib‐containing regimen administration to the first event (death for OS and death, progression or institution of next line of therapy for PFS). Time to next treatment was calculated from the date of bortezomib‐containing therapy initiation until the date of commencement of subsequent therapy, with death a competing risk. Patients who had not experienced an event were censored at last follow‐up. OS, PFS and time to next treatment were estimated using Kaplan–Meier methods.

## 3 RESULTS AND DISCUSSION

**FIGURE 1:**
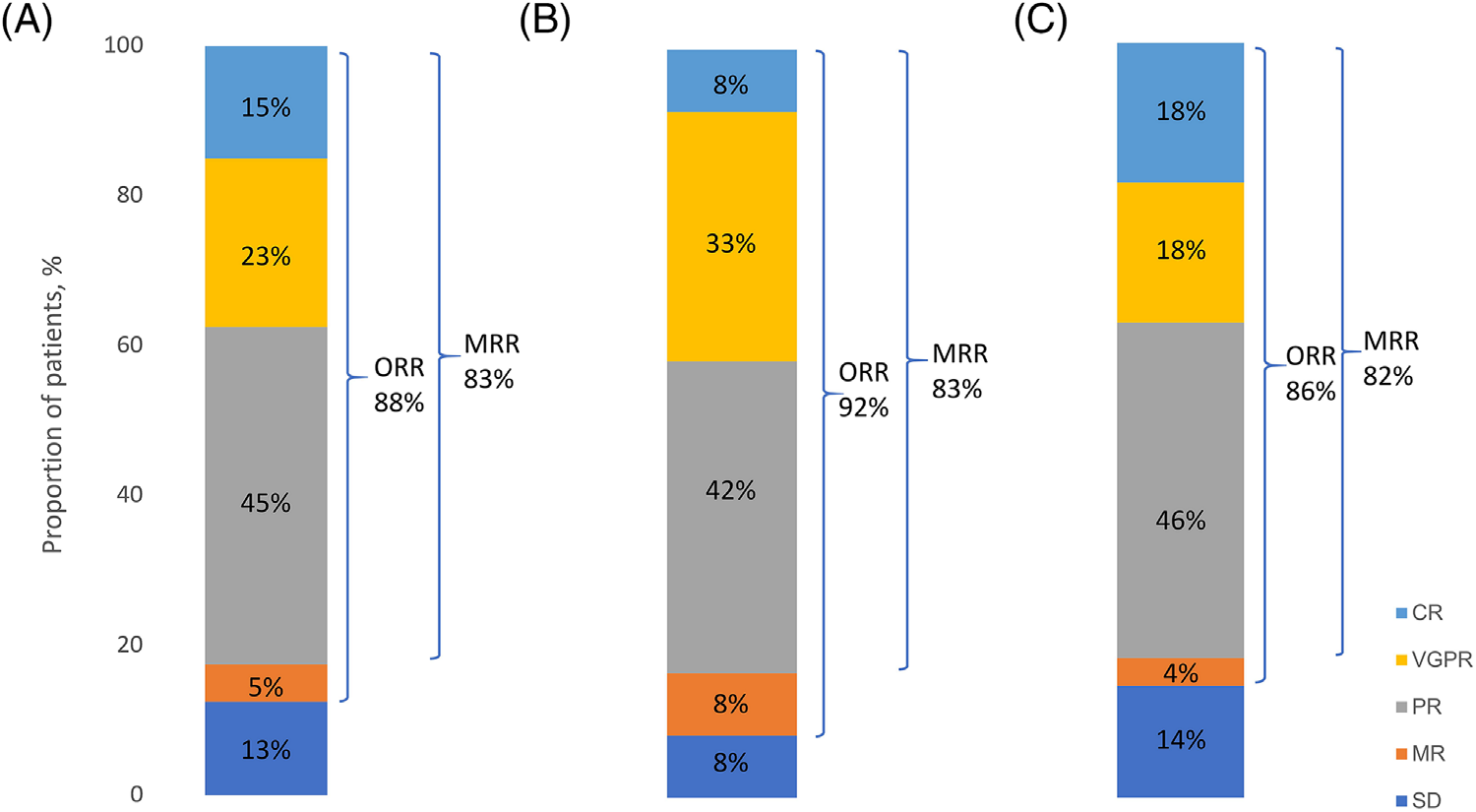
**Response rates to bortezomib‐containing regimens**. (A) Entire cohort. (B) Frontline. (C) Relapse. CR, complete response; VGPR, very good partial response; PR, partial response; MR, minor response; SD, stable disease; ORR, overall response rate; MRR: major haematological response (≥PR)

Forty‐one patients were identified (29 male, 12 female). Thirty‐nine patients had one subcutaneous bortezomib‐containing regimen, and two patients had >1 subcutaneous bortezomib‐containing regimen, totalling 43 regimens administered in all. Baseline characteristics and responses are outlined in Table 1 and Figure 1, respectively. At bortezomib initiation, the median age was 60 years (range 37–87; 20% age > 70 years), performance status was 1 (range 0–2), *MYD88^L265P^* was present in 77% (17/22) and *CXCR4* mutated in 27% (4/15). The majority were treated with a bortezomib‐containing regimen at relapse (71%; 29/41), whilst 29% (12/41) were treated frontline. Of patients treated at relapse, the median prior lines of therapy were 3 (range 1–7). Overall, the time to bortezomib‐containing treatment was 66 months (range 0–422) from WM diagnosis. Most patients had prior alkylator exposure. Twenty four per cent (10/41) of the entire cohort were refractory or intolerant to BTKi.

**TABLE 1:** Baseline characteristics and bortezomib‐containing regimens

*n* (% or range)	All *n* = 44	Frontline *n* = 12	Relapse *n* = 32
**Age**, years*	60 (37–87)	55 (37–87)	66 (46–82)
**Gender** *			
Male	29 (71)	9 (75)	20 (69)
Female	12 (29)	3 (25)	9 (31)
**Mutational status**			
*MYD88^L265P^* present	17/22 (77)	7/8 (88)	10/14 (71)
*CXCR4* mutated	4/15 (27)	2/3 (67)	2/12 (17)
**Prior lines of therapy**	**–**	–	
1			6 (19)
2			9 (28)
3			8 (25)
>3			9 (28)
**Prior drug exposure**	‐		
RCHOP			12 (38)
DRC			11 (34)
BTKi			10 (31)
Bendamustine			8 (25)
Chlorambucil			6 (19)
Pembrolizumab			4 (13)
Cladrabine			3 (9)
FCR			3 (9)
ASCT			2 (6)
Venetoclax			1 (3)
**M‐protein**, g/l	30 (4–60)	41 (7–52)	24 (4–60)
**Haemoglobin**, g/l	95 (70–148)	97 (70–103)	93 (71–148)
**Platelets**, x 10^9^/l	205 (0–445)	205 (41–301)	203 (10–445)
**Bortezomib‐containing regimen**			
BDR	27 (62)	11 (92)	16 (50)
Bortezomib‐dexamethasone	8 (18)	0	8 (25)
VCD ± Rituximab	6 (14)	1 (8)	5 (16)
Daratumumab‐bortezomib‐dexamethasone	1 (2)	0	1 (3)
Bortezomib‐DRC	1 (2)	0	1 (3)
Bortezomib‐bendamustine‐ofatumumab	1 (2)	0	1 (3)
**Toxicity**	*n* = 41	*n* = 12	*n* = 29
**Neuropathy**			
Grade 1–2	10 (24)	3 (25)	7 (24)
**Gastrointestinal**			
Grade 1–2	2 (5)	1 (8)	1 (3)
Grade 4	1 (2)	0	1 (3)
**Infection**			
Grade 1–2	2 (5)	0	2 (7)
Grade 3	3 (7)	0	3 (10)

Abbreviations: ASCT, autologous stem cell transplant; BDR, bortezomib dexamethasone rituximab; DRC, dexamethasone rituximab cyclophosphamide; FCR, fludarabine cyclophosphamide rituximab; M‐protein, monoclonal protein; RCHOP, rituximab cyclophosphamide doxorubicin vincristine prednisolone; VCD, bortezomib cyclophosphamide dexamethasone.

* *n* = 41.

** 44 regimens include two patients with >1 bortezomib‐containing regimen (treated for relapse disease).

At bortezomib initiation, the median M‐protein was 30 g/l (range 5–60) with bone marrow LPL infiltration 70% (range 5–100), haemoglobin was 95 g/l (range 70–148, 96% < 115 g/l), and platelet count was 205 × 10^9^/l (range 10–380, 30% < 100 × 10^9^/l).

Bortezomib‐dexamethasone‐rituximab was the most frequently delivered regimen (62%), followed by bortezomib‐dexamethasone (18%). A median of six cycles (range 1–12) were delivered; 56% (15/27) received a dose of 1.3 mg/m^2^, and 44% (12/27) received a dose of 1.6 mg/m^2^ at weekly doses for 3–4 doses per cycle for up to 10 cycles and monthly thereafter. No patients had twice weekly administration.

Toxicity was assessable in 39/41 regimens. Grade 1–2 neuropathy occurred in 24% (10/41) but did not result in treatment cessation in any case. No grade 3–4 neuropathy was observed. Grade 1–3 infection occurred in 12% (5/41: cytomegalovirus colitis, *n* = 1; culture‐positive bacterial infections, *n* = 2; suspected *n* = 2), exclusively in those treated for relapsed disease. Gastrointestinal disturbance occurred in 7% (3/41); one patient required hospital admission with grade 4 diarrhoea. Seventeen per cent (7/41) required bortezomib dose reduction, due to grade 1–2 neuropathy (*n* = 4), diarrhoea (*n* = 1), microangiopathic haemolytic anaemia (*n* = 1) and musculoskeletal pain (*n* = 1). One patient discontinued treatment due to gastrointestinal toxicity (grade 4 diarrhoea), and in all others that required dose reduction, adverse events stabilised or reversed (*n* = 3).

For the entire cohort, the overall response rate (major and minor response) was 88%. Best major response rate (≥partial response, PR) was 83% at a median time of 4 months (range 1–23) from start of therapy. Major response rate was comparable in those receiving a bortezomib dose of 1.3 mg/m^2^ compared with 1.6 mg/m^2^ (93% [13/14] vs. 67% [8/12], *p* = 0.15). Ten patients had prior covalent or non‐covalent BTKi use (ibrutinib *n* = 6; pirtobrutinib and ibrutinib *n* = 2; acalabrutinib *n* = 2). Of these, 1 achieved complete response, 1 very good partial response, 4 PR, 1 minimal response, 1 stable disease, 2 too early to assess. Major response rate was comparable in those with prior BTKi compared with those without (75% [6/8] vs. 84% [27/32], *p* = 0.61).

For the entire cohort, the median bone marrow infiltration after treatment was 10% (range 0–85). The median follow‐up after bortezomib administration was 34 months (range 0–131). Estimated 2‐year OS and PFS were 90% (95% confidence interval [CI] 73–96) and 76% (95% CI 56–87), respectively. Median OS was not reached, median PFS was 36 months, and median time to next treatment was 66 months. Two‐year OS for frontline and relapse WM was 100% (95% CI 100) and 87% (95% CI 64–95), respectively, and 2‐year PFS for frontline and relapsed WM was 88% (95% CI 39–98) and 71% (95% CI 46–86), respectively. Two patients died during treatment: one due to infection (COVID) and one due to high‐grade lymphoma transformation after achieving initial PR. No patients developed secondary myelodysplastic syndrome. Of patients that relapsed post‐bortezomib therapy, BTKi was employed in four with haematological responses: 2/12 treated with bortezomib at frontline achieved a VGPR and PR, respectively, after BTKi; 2/29 treated with bortezomib at relapse, achieved a CR and VGPR, respectively after BTKi.

Our data have inherent limitations due to its retrospective nature. Baseline prognostic scoring (International Prognostic Score System for WM) and molecular studies (*MYD88* or *CXCR4* status) were not complete in all patients and therefore may have limited analysis of potential confounders. At present there are no strong prospective data to support treatment decisions based on mutational status; however collecting these data will be essential to inform the role of genomic assessment. Toxicity was low in our cohort; however as a non‐controlled study, it was prone to potential selection bias for a fit group of patients. The small sample size in our cohort precluded meaningful subgroup analysis, and this emphasises the more data in this area.

Management strategies for WM have evolved with the introduction of targeted therapy including new classes of BTKi and potential cellular therapies. Limited duration of therapy, manageable toxicity and effective deep responses remain lacking particularly in the relapsed population. Bortezomib may be utilised in fixed‐duration drug combinations (e.g., bortezomib‐dexamethasone‐rituximab) or for longer durations maintenance therapy (e.g., bortezomib‐dexamethasone).

With the increasing use of BTKi therapy, patients who are intolerant or refractory will require careful selection of alternative strategies and enrolment in clinical trials. Beyond traditional alkylators and bendamustine, novel therapies may be considered. Anti‐CD38 monotherapy, daratumumab, has been employed in a phase 2 study with 13 patient, with an ORR of 23% and median PFS of only 2 months in previously treated patients with WM [8]. Active and upcoming clinical trials include the use of the PD1 inhibitor pembrolizumab (phase 2, NCT03630042), BTKi in combination with BCL2 inhibitors (phase 1, NCT05024045), radiopharmaceutical approaches with radiolabelled iopofosine (phase 2, NCT02952508) and CAR‐T therapy (ZUMA‐25). Recruitment onto clinical trials may be limited by access however bortezomib may act as a bridge to these therapies.

We demonstrate efficacy and tolerability of bortezomib at frontline and in multiply relapsed patients with WM. Bortezomib‐containing regimens are highly active with a 2‐year OS 90% and PFS 76% and median time to next treatment of 66 months. It is an effective therapy even in those multiply relapsed (up to seven prior lines in our cohort) with heavy marrow infiltration and BTKi failure. Gastrointestinal and neurotoxicity may be manageable with subcutaneous bortezomib formulation and appropriate dose reductions. Our toxicity rates were lower than previous reports particularly of intravenous bortezomib administration with grade ≥2 neuropathy above 60% [1, 7]; we observed no grade 3–4 neurotoxicity. Monitoring for these side effects may abrogate potential toxicity. Treatment discontinuations are rare in this real‐world cohort, suggesting a manageable safety profile. Responses are durable, and the prolonged time to next treatment in this study reflects that despite a proportion meeting clinical progression (>25% in IgM level), patients may be asymptomatic and not require treatment. Bortezomib should be considered as a treatment modality particularly in those who are refractory to BTKi.

## AUTHOR CONTRIBUTIONS

JK and EU collected the data, performed analysis and wrote the manuscript. RB, KT and IP performed laboratory analysis. AR, RG, CK and SD reviewed the data and manuscript.

## CONFLICT OF INTEREST

SDS reports speaker fees and research funding from Janssen, BeiGene and Sanofi. JK, EU, RB, KT, AR, RG, IP and CK have no conflict of interest to declare.

## Data Availability

The data that support the findings of this study are available upon request from the corresponding author. The data are not publicly available due to privacy or ethical restrictions. N/a cd_value_code=text
